# Recipe for a Healthy Gut: Intake of Unpasteurised Milk Is Associated with Increased *Lactobacillus* Abundance in the Human Gut Microbiome

**DOI:** 10.3390/nu12051468

**Published:** 2020-05-19

**Authors:** Mary I. Butler, Thomaz F. S. Bastiaanssen, Caitriona Long-Smith, Kirsten Berding, Sabrina Morkl, Anne-Marie Cusack, Conall Strain, Kizkitza Busca, Penny Porteous-Allen, Marcus J. Claesson, Catherine Stanton, John F. Cryan, Darina Allen, Timothy G. Dinan

**Affiliations:** 1 APC Microbiome Ireland, University College Cork, T12 YN60 Cork, Ireland; thomaz.bastiaanssen@ucc.ie (T.F.S.B.); c.longsmith@ucc.ie (C.L.-S.); kirsten.berdingharold@ucc.ie (K.B.); sabrina.moerkl@medunigraz.at (S.M.); am.cusack@ucc.ie (A.-M.C.); conall.strain@teagasc.ie (C.S.); kizkitza80@hotmail.com (K.B.); m.claesson@ucc.ie (M.J.C.); catherine.stanton@ucc.ie (C.S.); j.cryan@ucc.ie (J.F.C.); t.dinan@ucc.ie (T.G.D.); 2 Department of Psychiatry, University College Cork, T12 YN60 Cork, Ireland; 3 Department of Anatomy and Neuroscience, University College Cork, T12 YN60 Cork, Ireland; 4 Department of Psychiatry and Psychotherapeutic Medicine, Medical University of Graz, 8036 Graz, Austria; 5 Teagasc Food Research Programme, Moorepark, Fermoy, Co. Cork, T12 YN60 Cork, Ireland; 6 Ballymaloe Cookery School, Organic Farm and Gardens, Shanagarry, Co. Cork, T12 YN60 Cork, Ireland; penny.porteous@gmail.com (P.P.-A.); darina@cookingisfun.ie (D.A.); 7 School of Microbiology, University College Cork, T12 YN60 Cork, Ireland

**Keywords:** microbiome-gut-brain axis, microbiota, probiotic, *Lactobacillus*, unpasteurised, raw, milk, dairy

## Abstract

Introduction: The gut microbiota plays a role in gut–brain communication and can influence psychological functioning. Diet is one of the major determinants of gut microbiota composition. The impact of unpasteurised dairy products on the microbiota is unknown. In this observational study, we investigated the effect of a dietary change involving intake of unpasteurised dairy on gut microbiome composition and psychological status in participants undertaking a residential 12-week cookery course on an organic farm. Methods: Twenty-four participants completed the study. The majority of food consumed during their stay originated from the organic farm itself and included unpasteurised milk and dairy products. At the beginning and end of the course, participants provided faecal samples and completed self-report questionnaires on a variety of parameters including mood, anxiety and sleep. Nutrient intake was monitored with a food frequency questionnaire. Gut microbiota analysis was performed with 16S rRNA gene sequencing. Additionally, faecal short chain fatty acids (SCFAs) were measured. Results: Relative abundance of the genus *Lactobacillus* increased significantly between pre- and post-course time points. This increase was associated with participants intake of unpasteurised milk and dairy products. An increase in the faecal SCFA, valerate, was observed along with an increase in the functional richness of the microbiome profile, as determined by measuring the predictive neuroactive potential using a gut–brain module approach. Conclusions: While concerns in relation to safety need to be considered, intake of unpasteurised milk and dairy products appear to be associated with the growth of the probiotic bacterial genus, *Lactobacillus*, in the human gut. More research is needed on the effect of dietary changes on gut microbiome composition, in particular in relation to the promotion of bacterial genera, such as *Lactobacillus*, which are recognised as being beneficial for a range of physical and mental health outcomes.

## 1. Introduction

A growing body of evidence over the past decade has demonstrated the importance of the gut microbiome in all aspects of physical and mental health. While it is still unclear what exactly constitutes a ‘healthy’ gut microbiome, certain bacterial groups have been strongly associated with better health outcomes. *Lactobacillus* is one of the foremost genera considered to have probiotic properties [1]. A probiotic is defined as a live microorganism which, when administered in adequate amounts, confers a health benefit on the host [2]. The word ‘*psychobiotic*’ is an expansion of this term and describes an organism that has been proven to be beneficial in relation to psychological functioning [3]. There have been a wide variety of studies undertaken in recent years that have demonstrated the benefit of a *Lactobacillus* probiotic, both mono- and multi-strain, for improving a range of health outcomes, including obesity [4], diabetes [5], liver disease [6], cardiovascular disease [7], gastrointestinal conditions [8] and neuropsychiatric disorders, such as depression, anxiety and autism [9].

A key present-day challenge involves identifying the most effective ways of maintaining a healthy gut microbiome and promoting the growth of probiotic bacteria. While commercial probiotic products are widely available, there are concerns in relation to regulation, quality control, efficacy and cost [10]. Dietary intake is one of the main factors regulating gut microbiome composition and food-based interventions can be tailored to each individual to modify their bacterial profile [11]. While unravelling the diet–microbiome relationship is a formidable task given the many confounding factors, attempts to do so have been made over the past decade. Gut microbiome profile has been shown to be distinctly different in those living in rural areas with a traditional diet in comparison to urban-based westernised populations [12,13,14]. Even when one accounts for contributions of human genetic and geographical factors between populations, subsistence methods and diet significantly impact gut microbiota composition [15]. It is hypothesised that a ‘microbiota insufficiency syndrome’ has resulted from modern lifestyle with its highly processed diets, overuse of antibiotics and increased sanitation and that the ‘industrialised’ microbiota may be a major contributing factor in the rise of many non-communicable chronic diseases in westernised societies [16]. Even as one moves from looking at the early ancestral microbiota to more recent times, significant changes in lifestyle have continued until relatively recently. Ireland, as with many countries in the developed world, was a predominantly agrarian society up until the mid-late 20th century. In 1966, over 30% of the workforce were employed in agriculture, with this figure estimated at less than 5% in 2016 [17]. Consumption of unpasteurised milk was a common part of the diet of those living on farms and epidemiological studies suggest that it may have played a protective role against the development of allergies and atopic diseases [18].

Despite food safety concerns, the consumption of unpasteurised milk appears to be growing in popularity [19,20]. To our knowledge, there are no studies exploring the impact of unpasteurised milk intake on the gut microbiome. In this observational study, we investigated the effect of a dietary change involving the intake of unpasteurised milk on gut microbiota composition, metabolites and psychological status in 24 participants undertaking a residential, farm-based, 12-week cookery course. Our centre had previously published a study [21] on the microbiota composition of unpasteurised milk taken from Irish cows, which would thus be representative of the expected microbiota composition of the raw milk that would be consumed by participants in our study. Given the reported high proportion of viable probiotic bacteria such as *Lactobacillus* (and other LAB such as *Lactococcus* and *Leuconostoc*), along with the fact that Lactobacillus are considered intrinsically resistant to gastric acid [22], we hypothesised that a dietary change involving raw milk consumption would alter the gut microbiome of participants with a potential differential increase in the relative abundance of these probiotic bacterial groups.

## 2. Methods

### 2.1. Study Site and Subjects

Ballymaloe Cookery School, Organic Farm and Gardens is located in East Cork, Ireland, and runs bi-annual 12-week residential courses where students live on-site, learn about organic farming methods and undertake intensive cookery classes. The majority of food consumed by participants during their stay originates from the organic farm itself or consists of high-quality, locally-sourced produce. The farm has a small herd of Jersey cows whose milk is used in its raw unpasteurised state for direct consumption, cooking and the production of other dairy products including cream, butter, cheese and yoghurt. There is an emphasis on eating, and cooking with, local seasonal fruit and vegetables, the vast majority of which is organic. Meat and fish are also locally sourced and, for the most part, organic.

Approval of the study protocol was granted by the Clinical Research Ethics Committee of the Cork Teaching Hospitals (Protocol number DOP001) and conducted following the ICH Guidelines on Good Clinical Practice, and the Declaration of Helsinki. Written informed consent was obtained from all subjects before study procedures were conducted. Course participants were emailed in advance informing them of the study and a short talk on the gut microbiome was given at an introductory session prior to commencement of the course. In order to be eligible for the study, participants had to be between the age of 18–65 years and be generally healthy, with no chronic or current, physical or mental illness. Exclusion criteria included the use of medications that were likely to interfere with the objectives of the study (including any psychotropic medications) as well as intake of antibiotics, probiotics or prebiotics within the month prior to commencement of the study.

### 2.2. Subject Metadata

Demographic data was collected for each individual including information on age, sex and race. Weight and height were measured and used to calculate body mass index (BMI). Information in relation to medical and psychiatric history, along with medication use, was also obtained at interview. At the beginning and end of the 12-week course participants completed self-report questionnaires on a variety of parameters including mood, anxiety, sleep, exercise (PSS, HADS, PSQI, International Physical Activity Questionnaire (IPAQ)).

### 2.3. Diet Quantification

To monitor nutrient intake, participants completed the self-administered 152-item SLAN-06 (Survey of Lifestyle, Attitudes and Nutrition in Ireland) food frequency questionnaire (FFQ) [23], which is validated to be used in an Irish population. An additional eight food items as well as questions about type and frequency of milk, salt and fried food consumption were added. These items are included in the EPIC Norfolk questionnaire [24] from which the SLAN-06 FFQ was adapted. An extra section was added to the FFQ by the authors to quantify the intake of unpasteurised milk and dairy products before and during the course, as this information would not otherwise have been captured. These extra questions followed the same response format as the other food items.

Participants were asked to estimate the frequency with which they consumed a specified portion size of each of the foods listed over the preceding month. The FFQ has nine possible responses ranging from “never or less than once per month” to “6+ per day”. Participants completed the FFQ before and after the stay at Ballymaloe. The FFQs were analysed for nutrient intake using the FETA software [25].

The 160 foods items were grouped into 29 food groups (e.g., fruits, vegetables, grains, sweets) using methods similar to those described in previous studies of dietary patterns [26]. To estimate the number of servings of any food group, each response was converted to the corresponding frequency factor and summed over all the food items to get the average servings of a specific food group per day. Intake of unpasteurised milk and dairy products was analysed in a similar way.

### 2.4. Faecal Sample Collection and 16S rRNA Gene Sequencing and Processing

Faecal samples were collected at the beginning and end of the 12-week period in disposable plastic containers with a Thermo Scientific™ Oxoid AnaeroGen 2.5 L Sachet in situ to generate anaerobic conditions within the container. Participants were instructed to keep the sample containers in a refrigerator at 4 °C. Samples were collected and transferred to a −80 °C freezer within 12 h.

DNA was extracted using the DNA Fast Stool DNA extraction kit (Qiagen, Hilton, Germany) using the protocol for Gram positive bacteria and including an additional bead beating step at the beginning of the procedure. DNA was quantified using the Qubit High Sensitivity Kit (Life Technologies, Carlsbad, CA, USA), standardised and then used as a template for PCR. 16S metagenomic libraries were prepared using primers to amplify the V3–V4 region of the bacterial 16S rRNA gene, with Illumina adaptors incorporated as described in the Illumina 16S Metagenomic Library Preparation guide. Following index PCR and purification, the products were quantified using the Qubit high sensitivity DNA kit (Life Technologies) and pooled equimolarly. The pooled libraries were assessed using an Agilent high sensitivity DNA kit and examined by quantitative PCR (qPCR) using the Kapa Quantification kit for Illumina (Kapa Biosystems, Wilmington, MA, USA) according to the manufacturer’s guidelines. Libraries were then diluted and denatured following Illumina guidelines and sequenced (2 × 300 bp) on the Illumina MiSeq platform.

### 2.5. Sequence Table Generation

Three hundred base pair paired-end reads were prefiltered based on a quality score threshold of >28 and trimmed, filtered for quality and chimaeras using the DADA2 library in R (version 3.6.3, Vienna, Austria) [27]. Taxonomy was assigned with DADA2 against the SILVA SSURef database release v132. Parameters as recommended in the DADA2 manual were adhered to unless mentioned otherwise. ASVs were cut off at genus level, those that were unknown on the genus level were not considered in downstream analysis, as were genera that were only detected as non-zero in five per cent or fewer of total samples.

### 2.6. Short Chain Fatty Acid (SCFA) Measurements

The concentration of SCFAs, acetate, propionate, Iso-butyrate, butyrate, Iso-valerate, and valerate were analysed by gas chromatography flame ionisation detection (GC-FID) using a Varian 3800 GC system, fitted with a guard column (Restek) connected to an Agilent DB-FFAP column (30 mL × 0.3 m ID × 0.25 μm df) and a flame ionisation detector with a CP-8400 auto-sampler.

### 2.7. Statistical Analysis

Statistical analysis for changes in dietary measures was performed using SPSS Statistical Packages version 25 (SPSS, Inc., Chicago, IL, USA). Normality of outcome measures were assessed using Shapiro Wilk’s test of normality. Differences in nutrient and food group intake pre- and post-course participation were analysed using Student’s t-test or the non-parametric Wilcoxon Rank sum test.

Microbiome data-handling was done in R (version 3.6) with the Rstudio GUI (version 1.2.1555). In all cases, the iNEXT library was used to calculate alpha diversity [28].

Principal component analysis (PCA) was performed on centred-log ratio transformed (clr) values using the ALDEx2 library [29]. Number of permutations was always set to 1000. Aitchison distance was used as a distance metric for beta-diversity. Piphillin [30] was used for functional inference from 16S rRNA gene sequences of stool samples in the form of Kyoto Encyclopedia of Genes and Genomes (KEGG) orthologues. Gut–brain modules were calculated using the R version of the Gomixer tool [31]. Differential abundance of microbes between groups was assessed using the ALDEx2 library. As part of testing for correlations between microbial abundance and metadata, skadi, an implementation of jackknifing and Grubb’s test, was used to assess the reliability of the data and detect outliers (R scripts available online, https://github.com/thomazbastiaanssen/Tjazi; [32]). Correlation was assessed using Spearmans’s rank correlation coefficient. The relationship between categorical variables was assessed using Pearson’s Chi-squared test. For datasets in which the condition of normality was violated, the non-parametric Kruskal–Wallis test was used and post-hoc analysis was done using the Wilcoxon test. A *p*-value of <0.05 was deemed significant. To correct for multiple testing in tests correlating volatility and specific microbiota, KEGG orthologues or pathways, the *Q*-value post-hoc procedure was performed with a *q*-value of 0.1 as a cut-off [33].

## 3. Results

### 3.1. Participant Characteristics

A total of 62 participants who were completing the 12-week course between May and July 2018 were informed about the study. Twenty-eight participants volunteered and underwent screening. Two were excluded; one had a chronic gastrointestinal disorder and another had taken antibiotics in the previous month. Twenty-six participants were enrolled with 24 (13 females, 11 males) completing the study; 2 failed to provide faecal samples. Of note, subject metadata and faecal samples were collected within the first three days of the course and again at week 11. The final week of the course (week 12) involved several examinations for students, and thus, the associated increased stress during this week may have had the potential to influence findings. Baseline characteristics of participants, including age, body mass index (BMI), smoking status, sleep quality and exercise levels are shown in Table 1.

### 3.2. Changes in Diet

Based on food frequency questionnaire (FFQ) analysis (Table 2 and Table 3), there was no change in total calorie intake during the course. In terms of macronutrient intake, protein and carbohydrate intake remained unchanged, and though total fat consumption increased, this change did not reach statistical significance (mean increase (g) from 94 ± 35 to 128 ± 66, *p* = 0.08). With regards to micronutrients, Vitamin A (µg) intake increased significantly (715 ± 577 to 1505 ± 975, *p* = 0.005) as did Vitamin B12 (µg) (7.8 ± 3.6 to 11 ± 5.8, *p* = 0.04). Although intake of fruit reduced slightly (2.02 ± 1.2 to 1.38 ± 0.84, *p* = 0.04), consumption of vegetables and wholegrains did not change, nor did intake of alcohol or unhealthy foods such as sweets or snacks.

Participants intake of milk and dairy products are summarised in Table 4. In relation to participants overall intake of milk, this did not change during the course (mean increase from 177 ± 120 mls to 192 ± 134 mls, *p* = 0.60). However, a switch to unpasteurised milk was evident for most participants. Only one of 24 (4%) participants reported consuming unpasteurised milk prior to commencing the residential course while 23 participants (96%) reported its consumption during the course (mean increase from 23 ± 116 mls to 239 ± 51 mls, *p* < 0.0001). Pre-course, 3 participants consumed skimmed milk, 7 semi-skimmed, 11 whole and 3 non-specific and post-course only one participant consumed semi-skimmed while the remaining participants consumed whole milk, consistent with unpasteurised milk intake. Total intake of dairy products (cream, yoghurt, dairy desserts, cheese; salad cream or mayonnaise, butter, cottage cheese) did increase slightly though not to a statistically significant level (mean increase from 2.24 ± 1.23 daily servings to 3.35 ± 3.16, *p* = 0.07). Two participants (8%) reported the intake of unpasteurised dairy products prior to the course whereas 21 (87.5%) consumed these products during the course (mean increase from 0.01 ± 0.04 servings per day to 1.2 ± 1.4, *p* < 0.0001).

### 3.3. Change in Microbiome Composition

**Figure 1:**
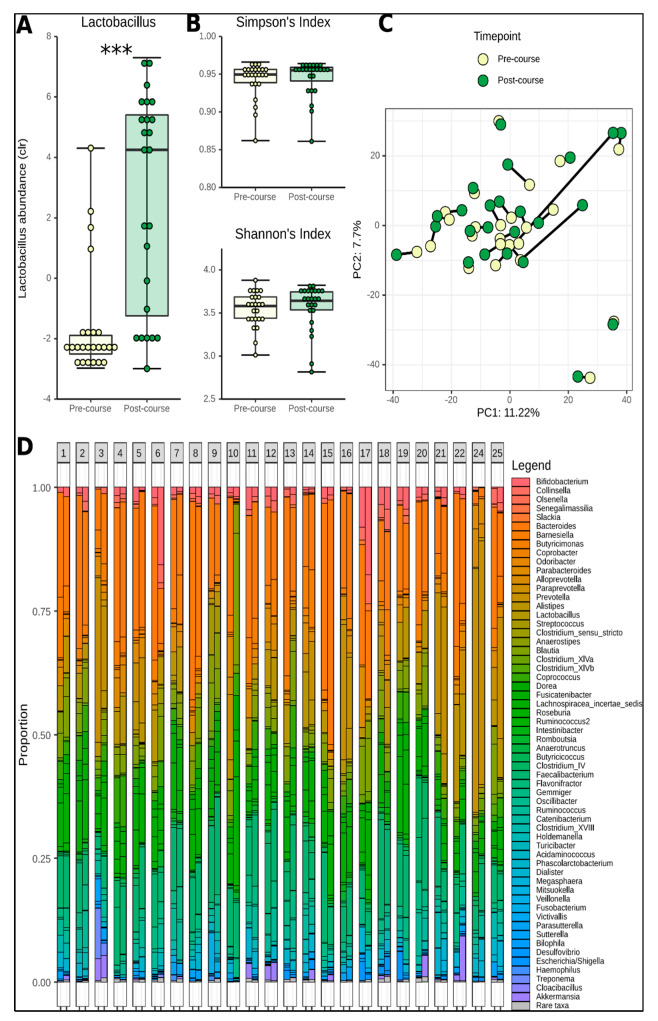
(**A**) Relative abundance of *Lactobacillus* at pre-course and post-course time points. (**B**) Alpha diversity of gut microbiome at pre-course and post-course time points. (**C**) Beta diversity of gut microbiome at pre-course and post-course time points. (**D**) Relative abundance of genus-level taxa for each participant. Each column represents one participant with pre-course taxa on the left and post-course taxa on the right. (Box plots: Body represents median and interquartile range, whiskers represent the extreme values).

We quantified the microbial diversity within each subject (α-diversity) before and after the course, and the difference between each subject’s pre-course and post-course gut microbiota (β-diversity). No significant differences were found in either α-diversity (Simpson; *p* = 0.41, Shannon; *p* = 0.26) or β-diversity (*p* = 0.998) (Figure 1B,C). No differences were found between males and females.

Analysis of the differential relative abundance of bacterial taxonomic groups revealed a total of 578 amplicon sequence variants (ASVs) within our samples (Figure 1D). Undirected pairwise analysis of all ASVs, (Wilcoxon signed rank test, allowing for Storey’s q-value post-hoc correction) revealed only one ASV which changed significantly between pre-course and post-course time points. This ASV corresponded to the genus *Lactobacillus*, which increased significantly (*p* = 0.0003728; q = 0.0498) (Figure 1A). Identification of ASVs at a species level was not possible. We subsequently performed a directed search in relation to other lactic acid bacteria (LAB), a dominant population in bovine milk prior to pasteurisation based on what was previously reported in literature on the subject [21]. On the genus level, the relative abundance of *Leuconostoc* (*p* = 0.09) and *Enterococcus* (*p* = 0.14) did not change but that of *Lactococcus* increased significantly (*p* = 0.01; q = 0.106). Other major components of unpasteurised milk include *Pseudomonas* and *Acinetobacter* [21]. We did not detect these genera in any of our samples.

**Figure 2:**
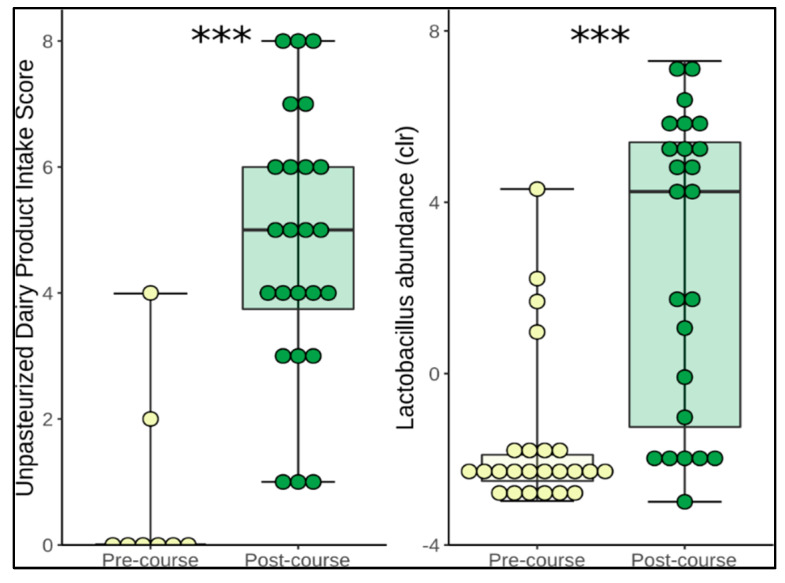
Box plots showing the change in combined unpasteurised dairy score and *Lactobacillus* abundance between pre- and post-course time points. (Body represents median and interquartile range, whiskers represent the extreme values; As some scores overlap, each participant is not visible as an individual point on the graph. Yellow represents pre-course scores and green represents post-course scores).

We directly tested for associations between changes in dietary components and changes in microbiome composition within each subject over time. We observed a positive correlation between Lactobacillus abundance and combined intake of unpasteurised milk and dairy products (Spearman correlation, r = 0.618, *p* = 0.0000027). Upon closer inspection, *Lactobacillus* abundance appeared to fall into two groups based on the centred log-ratio (clr) transformation of relative abundance scores of >2.5 or <2.5, prompting us to dichotomise the data for Pearson’s Chi-squared test. We defined these groups as low versus high *Lactobacillus* abundance and found a positive association between these two groups and the change in intake of unpasteurised milk and dairy products (combined score), binned into four groups based on the amount of portions consumed; 0–2, 3–4, 5–6 and 7–8. (Pearson’s Chi-squared, X-squared = 13.265, df = 3, *p*-value = 0.004096) (Figure 2). This association also held when looking at the relationship between *Lactobacillus* abundance and unpasteurised milk or unpasteurised dairy products individually. We analysed this *Lactobacillus* grouping against our other metadata (including age, sex, BMI, sleep, exercise and gastrointestinal parameters) but found no other factors associated with the high versus low split.

### 3.4. Functional Prediction and Application of Gut-Brain Modules

**Figure 3:**
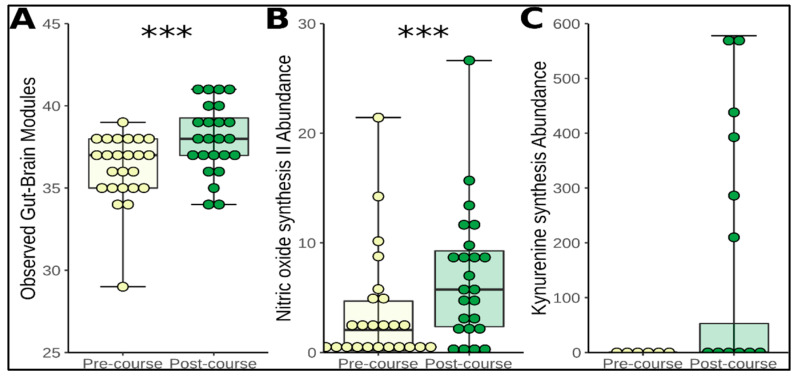
(**A**) Functional richness of microbiome, as measured by observed number of gut–brain modules (GBM) at pre-course and post-course time points. (**B**) Increase in abundance of GBM 026: Nitric oxide synthesis II (nitrite reductase) between pre- and post-course time points. (**C**) Increase in GBM 004: Kynurenine synthesis between pre- and post-course time points. (Box plots: Body represents median and interquartile range, whiskers represent the extreme values; As some scores overlap, each participant is not visible as an individual point on the graph. Yellow represents pre-course scores and green represents post-course scores).

Functional analysis of our microbiome data was performed using Piphillin [30] and further extended by subjecting our metagenomic data to a module-based analytical framework, which targets microbial pathways involved in microbiota-gut-brain communication, thus generating a prediction of the neuroactive potential of a microbiome sample [34]. Within our sample, we observed 43 of the 56 gut-brain modules (GBMs) described previously by the authors. In addition, we observed an increase in the functional richness of the microbiome profile, as determined by the number of gut–brain modules (GBMs) present (Wilcoxon signed rank test; mean increase of 1.79, *p* = 0.00087) following the 12-week course (Figure 3A). On analysis of the individual GBMs, one consistently increased significantly; GBM026: Nitric oxide synthesis II (nitrite reductase) (*p* = 0.001; q = 0.061) (Figure 3B). Notably, GBM004: Kynurenine synthesis was never found in participants pre-course, but was detected in 6 out of 24 participants post-course at very high levels. This observation did not pass the post-hoc correction (*p* = 0.036, q = 0.361) (Figure 3C). Functional alpha diversity, measured here by calculating the alpha diversity of the floored KEGG Orthologue tables generated by Piphillin, did not differ between pre- and post-course time points (chao1; *p* = 0.14, Simpson; *p* = 0.19, Shannon; *p* = 0.85).

### 3.5. Change in Microbiome Metabolites

Analysis of faecal short-chain fatty acids (SCFAs) revealed a significant increase in Valerate (*p* = 0.049) over the 12 weeks. Propionate also increased, although not to a statistically significant level (*p* = 0.08) while no change was observed in butyrate, iso-butyrate, iso-valerate or acetate (Table 5).

### 3.6. Change in Psychological Measures

There was no change in total scores on the Cohen’s Perceived Stress Scale (PSS), Hospital Anxiety and Depression Scale (HADS)-total score, HADS-anxiety subscale, HADS-depression subscale or Pittsburgh Sleep Quality Index (PSQI) sleep quality score between pre- and post-course time points. However, because our study involved a healthy population, baseline anxiety and stress scores were low and mood scores were within the normal range. (Table 6) Further analysis was considered taking into account baseline scores. The sample was dichotomised based on the median score of the above scales. Participants with higher baseline scores on the PSS showed a mean reduction of 4.42 points, whereas the rest of the participants reported a mean increase of 1 point (Mann–Whitney U test, *p* = 0.026) between pre-course and post course time points. Participants with higher anxiety scores than the median on the HADS-A also showed a statistically significant reduction compared to the rest of the participants (mean reduction of 2.11 vs. a mean increase of 2.25, Mann–Whitney U test, *p* = 0.004). We did not find any relationship between microbes and psychological scales. No differences were found between males and females.

## 4. Discussion

In this observational study, we investigated the effect of a dietary change on the gut microbiome of participants who undertook a 12-week residential cookery course on an organic farm, where the majority of food consumed and used for cooking, was locally-sourced, seasonal and produced using organic methods. Of particular interest was the use of unpasteurised milk and dairy products obtained from a small herd of Jersey cows on the farm. Most participants had not been using any unpasteurised dairy prior to the course and all used these products to some degree throughout their stay. We found that the main change in terms of microbiome composition was a dramatic increase in the participants’ *Lactobacilli* between pre-course and post-course faecal samples. This increase was strongly associated with the participants’ intake of unpasteurised milk and dairy products. In addition, a positive change was noted in relation to microbiome metabolites with an increase in valerate and, to a lesser extent not quite reaching statistical significance, propionate.

While administration of probiotics in the form of conventional pharmaceutical agents such as tablets or capsules is a common method, the majority of probiotics commercially available are in the form of food-based delivery systems, which use probiotic bacteria in their production or add these bacteria during the manufacturing process, for example, cheese, yoghurt or fermented drinks [35]. There are several problems associated with pharmaceutical and commercially-produced probiotic formulations. Firstly, the probiotic potential of bacteria is species and strain-specific but efficacy is often generalised across products in the current unregulated commercial probiotic market [36]. Secondly, there are many aspects of the manufacturing process of such products, which can alter the delivery of viable functional probiotic bacteria [37]. Because probiotic products are generally categorised as food supplements, they are subject to less stringent regulatory criteria and quality control processes with regard to microorganism specification, their numbers and functional properties [10]. Thirdly, there is a cost consideration when it comes to commercial probiotic products, which may place daily probiotic supplements out of the reach of many.

An alternative to consuming commercially-produced probiotic supplements for the maintenance of a healthy gut microbiome is to alter one’s diet. It is increasingly accepted that the ‘Western-diet’, characterised by highly-processed, low-fibre, high-sugar, high-fat foodstuffs has negative implications for health [38], which may be mediated by an unfavourable impact on the gut microbiome [39]. In contrast, adherence to a Mediterranean-style diet (characterised by high-level consumption of olive oil, fruit, nuts, vegetables, and cereals with moderate intake of fish and poultry) has been strongly associated with better physical [40] and mental [41] health outcomes, which again may be related to a beneficial impact on the gut microbiome and metabolome [42]. Gut microbiome composition can be rapidly and significantly altered by introducing dietary change [43] with the impact of food choices on the microbiome being highly individualised [11]. In this study, the key change in relation to dietary intake during the 12-week residential course was an increase in dairy products, which in this context were unpasteurised. This was a major change for our subjects, the vast majority of whom did not consume unpasteurised milk or dairy products prior to the course.

Cow’s milk is produced on a massive scale worldwide and has long played an important role in human nutrition [44]. Cow’s milk harbours a rich microbiota and typically contains a significant population of lactic acid bacteria (LAB) that includes *Lactococcus* (8.2 × 10^1^–1.4 × 10^4^ CFU/mL), *Streptococcus* (1.41 × 10^1^–1.5 × 10^4^ CFU/mL), *Lactobacillus* (1.0 × 10^2^–3.2 × 10^4^ CFU/mL), *Leuconostoc* (9.8 × 10^1^–2.5 × 10^3^ CFU/mL) and *Enterococcus* spp. (2.57 × 10^1^–1.58 × 10^3^ CFU/mL) [45]. Other organisms present in substantial proportions are *Pseudomonas* and *Acinetobacter*, so-called psychrotrophs which can flourish during cold storage conditions and typically cause milk spoilage [46]. Pasteurisation of milk gained widespread popularity in the early 1900s when cow’s milk was linked to the spread of disease epidemics such as tuberculosis, diphtheria, typhoid fever, scarlet fever, anthrax and cholera [47]. A recent Irish study, using molecular, culture-independent techniques, compared the microbial content of raw and pasteurised cow’s milk [21]. Authors reported that, although the bacterial diversity of the raw and pasteurised milk was similar, raw milk contained mostly viable cells whereas the cell population in pasteurised milk were predominantly nonviable. Thus, while pasteurised milk appeared to have a somewhat similar microbiome composition to that of the raw milk, any potential probiotic LAB would have been in a nonviable state. In this study, *Pseudomonas* and *Acinetobacter*, two major genera found in unpasteurised milk, were not detected by 16S rRNA analysis of the microbiomes of the participants, either pre or post treatment. This may be due to a selective filtering effect of the human immune system or physiological barriers such as gastric acid, which is known to act as such a filter [48,49].

The consumption of raw milk is growing in popularity, although there is some debate in relation to its purported benefits and concern about the potential dangers of contracting milk-borne illnesses if the raw milk is contaminated with human pathogens [50]. There is a strong suggestion from epidemiological literature that the consumption of unpasteurised cow’s milk or yoghurt by children living on farms or rural areas has a protective effect against the development of asthma, allergies and atopy, a finding that seems to be independent of other farm-related exposures [18]. In addition, raw milk is anecdotally reported to be beneficial for people with lactose intolerance [51]. This is thought to be due to the fact that raw milk contains high counts of LAB that produce lactase enzymes, which would otherwise be destroyed during pasteurisation. However, there is little research evidence to support these anecdotal claims and, in fact, one recent pilot randomised controlled trial (RCT) involving 16 adults with lactose malabsorption, failed to find any benefit of raw milk over pasteurised milk for gastrointestinal symptoms [52]. Despite this, in a survey of raw-milk consumers [53], over one-third of responders claimed to experience gastrointestinal discomfort from drinking pasteurised milk but no discomfort after drinking raw milk, although the vast majority of these people did not have a diagnosis of lactose intolerance. Another proposed benefit of raw milk is that it contains higher quantities of vitamins. A meta-analysis [54] reported that pasteurisation reduced the concentrations of Vitamin E, Vitamin B12, Vitamin B2, Vitamin C and folate. Of these vitamins, B2 is of most importance as bovine milk contributes significantly to the recommended daily intake whereas in the case of all the others, milk is not typically an important source. In relation to the human gut microbiome, we are unaware of any studies specifically examining the effect of raw milk consumption. However, a few studies have investigated the impact of pasteurised milk on the human microbiome. One cross-sectional study reported a differential oral microbiome based on high versus low (pasteurised) milk intake [55]. Another investigated the impact of whole milk supplementation on the gut microbiota and cardiometabolic biomarkers between lactose malabsorbers (LM) and absorbers (LA) [56]. Authors found that whole milk supplementation significantly altered the intestinal microbiota composition in LM, resulting in an increase in the phylum Actinobacteria along with increases in several genera; *Bifidobacterium*, *Anaerostipes* and *Blautia*. These changes occurred only in LM and not LA, suggesting that it was the increased lactose substrate reaching the colon that preferentially enhanced the growth of some microorganisms. In addition to pasteurisation, milk can be altered by skimming, which is currently a widespread procedure. Prior to the course, 10/24 of our participants reported consuming skimmed or semi-skimmed milk, while post-course, 23/24 participants consumed whole milk, reflecting the unpasteurised milk intake. Skimmed milk contains less fat than whole milk and thus also less fat-soluble vitamins such as A and E. However, regular unfortified milk is not a major contributor to a person’s recommended daily allowance of these vitamins [57] and despite the variable amounts in different milk types there does not appear to be a significant difference in their bioavailability [58]. Other micronutrients such as calcium, sodium and choline do not differ between skimmed and whole milk [59]. Therefore, we considered the skimmed versus whole milk type to be of limited consequence.

An obvious limitation of this study is the inherent potential for confounding given that, in addition to a change in diet, study participants experienced a change in environment. Disentangling the impact of diet and geographical environment on the gut microbiome, however, is a very difficult task. Several large scale studies have attempted to explore the differences in microbiome composition between industrialised Western urban dwellers and those living in traditional rural communities in South America and Africa, such as the Hadza hunter-gatherers of Tanzania [60], rural Papua New Guineans [61], children from the rural African village of Burkina Faso [14] and communities from Malawi and Amazonian Amerindians [12]. Although a rural setting will likely contribute to gut microbiome differences, these farming environments are intrinsically linked to variation in diet and it is difficult to separate the impact of the farm environment itself and the farm-related dietary patterns. If a move to a rural farming environment were to account for the changes in microbiome seen in our study, one could postulate that the changes would be consistent with the microbiome composition in rural dwellers from the above studies. This was not the case. While rural dwellers from PNG did have higher abundance of *Lactobacillus* than their urban counterparts [61], those from the other rural farming communities did not [14,60]. Obviously, the rural locations in the above studies were at the extreme end in relation to geographical location and traditional lifestyle and poorly comparable to the developed farm environment in which our participants were based. In a study more closely resembling our location, authors compared the microbiome of infants from farming and non-farming families in Wisconsin, United States, and again no differences in *Lactobacillus* or other LAB abundance were seen [62]. Furthermore, the changes in bacterial taxa in the microbiome of our subjects were consistent with those species found in unpasteurised milk, supporting our conclusion that this specific dietary change was driving the microbiome differences between pre- and post-course time points.

In this study we found that, during the 12-week course, the levels of the faecal SCFA valerate increased with a trend towards an increase in propionate. Straight-chain SCFAs (acetate, butyrate, propionate and valerate) are produced by the gut microbiota during the fermentation of partially and nondigestible polysaccharides whereas branched-chain SCFAs (isobutyrate and isovalerate) result from the metabolism of proteins [63]. SCFAs are thought to play a major role in the maintenance of gut and immune homeostasis [64] as well as in the gut-brain axis response to stress [65]. SCFA production can be stimulated by increasing dietary fibre intake [66] or protein consumption [67]. However, in our study, participants intake of fibre or protein did not change, and thus, it is proposed that increased valerate and propionate levels may have been secondary to increased abundance of *Lactobacilli*, which, along with other LAB, are known producers of SCFA [68]. Propionate has anti-inflammatory properties and has been shown to be of potential benefit across a range of disorders, including hypertension and cardiovascular disease [69], obesity [70] and hypercholesterolemia [71]. Valerate is a less well-known SCFA with limited research to date into its therapeutic potential. However, a recent study revealed that it also appears to have an immunomodulatory effect [72]. Interestingly, supplementation with *Lactobacillus acidophilus* increased the concentration of valerate in the caecum of chickens infected with *Clostridium perfringens* while reducing the infection-associated gut dysbiosis [73]. Valerate may also hold some translatable therapeutic value in the context of *Clostridium difficile* infection (CDI). Valerate was shown to be significantly reduced in the faecal samples of patients with recurrent CDI and recovered following successful treatment with FMT [74].

**Figure 4:**
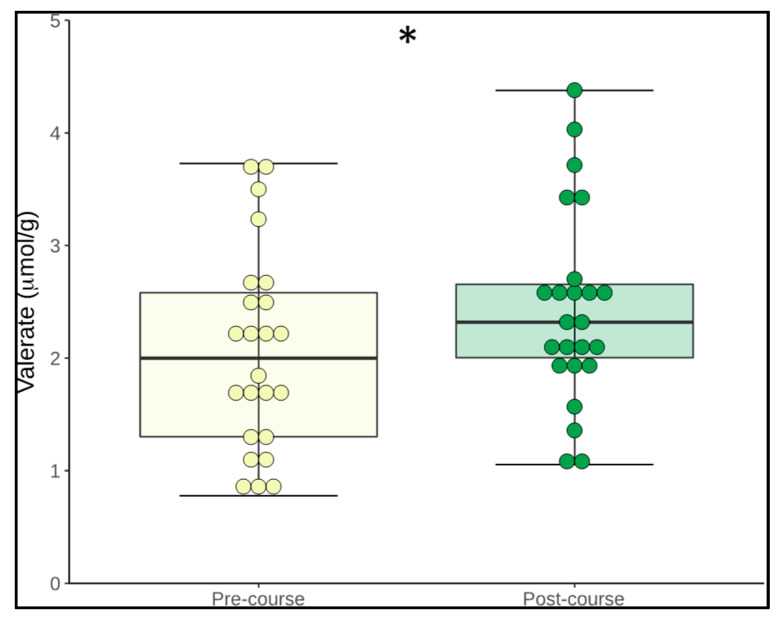
Concentration of Valerate at pre- and post-course time points. (Body represents median and interquartile range; whiskers represent the extreme values. Yellow represents pre-course scores and green represents post-course scores).

Changes in the functionality of the microbiome were assessed in the context of a recent study which facilitates analysis of the neuroactive potential of a microbiome sample [34]. Authors achieved this using a gut–brain-module (GBM) framework, which targets microbial pathways known to be involved in microbiota–gut–brain communication and have made this GBM catalogue available for use by other researchers (https://raeslab.org/software/gbms.html). When applying our predictive metagenomic data to this GBM catalogue, we found an increase in the functional richness of the microbiome profile, as determined by the number of GBMs present, following the 12-week course (Figure 4). Such a consistent general increase in GBMs without a significant increase in microbial alpha diversity goes somewhat against the intuition that a more diverse microbial ecosystem will necessarily display a higher functional diversity. More strikingly, the functional alpha diversity did not change during the course. GBMs represent a specific subset of microbiome function and are calculated using the values of specific KEGG Orthologues. A shift in microbial functions that specifically potentially impact the host brain without a corresponding general shift in microbial function detectible on the alpha diversity level shines light on the possibility that many more such specific shifts can occur undetected using current bioinformatics tools. Because of this, we call for a move away from general diversity and towards informed interrogation of specific functional changes in the microbiome as a readout.

One GBM changed significantly after post-hoc correction; ‘GBM026; Nitric oxide synthesis II (nitrite reductase)’. Several studies have demonstrated the ability of various *Lactobacillus* species to synthesise nitric oxide by nitrate reductase activity [75,76]. Nitric oxide is a complex and widespread signalling molecule that participates in virtually every organ system of the body. It is thought to play a role in the stress response and mood regulation [77] and may represent one mechanism by which *Lactobacilli* exert psychobiotic effects. The authors believe another GBM warrants discussing here, although its increase did not satisfy significance after post-hoc correction; ‘GBM004, Kynurenine synthesis’. This module was never detected in participants pre-course but was present in very high levels in 6 out of 24 participants post-course. This can be explained by the fact that the Kynurenine synthesis module requires two enzymatic steps. One of these was found in *Lactobacillus*, but the other one was not specific to a single microbe in this data set, but rather spread over several microbes and was only found in the six participants positive for MBG004. This finding conforms well with literature regarding the emergent biosynthetic capacity of the microbiome [78,79].

Although we found no direct correlation between *Lactobacillus* abundance and psychological measures, it is notable that stress and anxiety levels reduced significantly in those with higher baseline scores on the PSS and HADS-A. This is consistent with probiotic interventional trials in healthy populations, whereby an impact is often only seen in those with higher anxiety or depression scores at baseline [80,81]. Of course, there are many possible confounding factors when it comes to interpreting this reduction. Participants in this course had varying reasons for completing the course; for some, the purpose was to enhance or change their career options and, thus, possibly associated with stress; for others it was simply for leisure and viewed more as a holiday incorporating cookery classes. The change in environment and daily activity, the purpose of participation in the course and interaction with new people may all have contributed to psychological status. However, given the increasing evidence that the gut microbiome is an important node in gut–brain communication and that certain psychobiotics have anxiolytic effects, it is plausible to consider the possibility that the improvement in stress and anxiety may have been partially related to the increase in *Lactobacillus*. *Lactobacillus rhamnosus* (JB-1) has been shown to reduce anxiety behaviours in mice as well as altering central levels of gamma-aminobutyric acid [82], a key neurotransmitter in anxiety regulation. Several species of *Lactobacillus* have demonstrated the ability to reduce anxiety and stress levels in healthy subjects [83,84,85] as well as in patients with chronic fatigue syndrome [86] or laryngeal cancer [87].

There are several limitations to our study. Firstly, this was an observational study. While of course an RCT would be preferable, there are many challenges inherent in designing RCTs involving dietary interventions. It can be difficult to define appropriate control groups and effective blinding of participants and investigators is often extremely difficult [88]. In particular, it can be challenging to accomplish a high level of adherence with whole food, or dietary pattern, interventions. A major strength of our study in this regard was that our participants were based on-site for the entire duration of the study making it possible to ensure a consistency across individual diets, which would be difficult to achieve outside a residential setting. The potential confounding effect of the farm environment as an independent modulator of microbiome composition is addressed earlier in the discussion. Secondly, our sample size was quite small. However, previously published studies investigating the diet–microbiome relationship have involved participant numbers of ten or less [43,89] and have generally been of much shorter duration [11]. Another factor that may limit the generalizability of our study was that participants undertaking this course were interested in food and cooking. Thus, they were likely to have good nutritional knowledge and possibly healthier than average diets at baseline. A specific limitation in this regard was an absence of any information on the use of non-nutritive sweeteners (NNS). These are being increasingly used due to the concern about the negative health impact of high-sugar diets and have been shown to significantly, and generally negatively, impact the gut microbiome [90]. Finally, given the limitations of 16S rRNA gene sequencing we were unable to characterise organisms beyond the genus level. More accurate taxonomic classification would have been useful had shotgun metagenomic sequencing been performed. Despite these limitations, this is, to our knowledge, the first study to report on the potential impact of unpasteurised milk and dairy products on the human gut microbiome. 

## 5. Conclusions

While there are understandable concerns in relation to potential contamination and safety when it comes to unpasteurised milk, it is a rich source of probiotic bacteria. Abundances of *Lactobacilli* increased significantly following a 12-week dietary change, which involved the consumption of unpasteurised milk and dairy products by participants. *Lactococcus* abundance also increased, although to a lesser extent. These changes in microbiome composition were reflected by an increase in levels of the SCFA, valerate with an observed trend towards an increase of propionate, along with an increase in the predicted functional richness of the microbiome. While there was no overall change in psychological measures, stress and anxiety scores did decrease in those with higher baseline scores. Given the growing appreciation of the importance of a healthy gut microbiome and the limitations of commercial probiotic products, there is a need for further research into the effect of different dietary changes on the microbiome and subsequent mental health. In particular, the consumption of raw milk and other probiotic-rich fermented foods is growing in popularity and it is important that the effect of such products on the gut microbiome are investigated.

## Figures and Tables

**Table 1:** Baseline characteristics of participants.

	Pre-Course	Post-Course	*p*-Value
Number of participants	24		
Female; *n* (%)	13 (54)		
Mean age; *n* (range)	30.25 (18–59)		
Smoking status; *n* (%)	7 (29)		
BMI (kg/m)	24.87 (3.42)	25.33 (3.61)	0.1
Physical activity (as measured by IPAQ score)	4757.52 (4614.74)	3271.52 (7280.05)	0.32
Sleep quality (as measured by PSQI)	5.36 (2.87)	4.95 (2.91)	0.25
Bristol stool scale score	3.78 (1.085)	4.04 (0.706)	0.39
GI-Visual Analogue Scale; Satisfaction with bowel habit	38.37 (33.757)	27.29 (27.98)	0.25

BMI: Body Mass Index, GI: Gastrointestinal, IPAQ: International Physical Activity Questionnaire; PSQI: Pittsburgh Sleep Quality Index.

**Table 2:** Changes in dietary components between pre-course and post-course time points, obtained from analysis of food frequency questionnaires. (*p*-values reaching statistical significance are in bold and accompanied by an asterisk).

Nutrient	Recommended Daily Intake	Pre-Course	Post-Course	*p*-Value
Kilocalories	2000–2400 (males; depending on activity level)	2264 ± 1006	2723 ± 1494	0.47
Protein (g)	10%–35% of total energy	97 ± 40 (17%)	109 ± 57 (16%)	0.54
Fat (g)	20%–35% of total calories	94 ± 35 (37%)	128 ± 66 (42%)	0.08
Carbohydrate (g)	45%–65% of total calories	246 ± 158 (43%)	275 ± 178 (40%)	0.77
Alcohol (mL)	21 standard drinks (1/2 pint of beer, small glass of wine, one measure of spirits)	15 ± 13	14 ± 13	0.98
Monounsaturated fatty acids (g)	>12% of total energy	38 ± 17 (15%)	51 ± 27 (17%)	0.13
Polyunsaturated fatty acids (g)	>6% of total energy	16 ± 7 (6%)	22 ± 13 (7%)	0.20
Saturated fatty acids (g)	<10% of total energy	34 ± 13 (14%)	49 ± 25 (16%)	0.04 *
Cholesterol (mg)	300 mg	381 ± 173	469 ± 236	0.13
Total sugar (g)	<10% of total energy	115 ± 67 (20%)	125 ± 76 (18%)	0.81
Starch (g)		128 ± 96	147 ± 104	0.62
Fibre (g)	>25 g	19 ± 15	20 ± 15	0.88
Vitamin A (µg)	800 µg	715 ± 577	1505 ± 975	0.005 *
Thiamine (mg)	1.1 mg	1.8 ± 1.2	1.9 ± 1.2	0.87
Riboflavin (mg)	1.4 mg	2.3 ± 1.5	2.6 ± 1.7	0.45
Niacin (mg)	16 mg	27 ± 15	29 ± 19	1.00
Vitamin B6 (mg)	1.4 mg	3.1 ± 3.0	2.9 ± 1.9	0.81
Vitamin B12 (µg)	2.5 µg	7.8 ± 3.6	11 ± 5.8	0.04 *
Folate (µg)	200 µg	339 ± 284	328 ± 236	0.58
Vitamin C (mg)	80 mg	104 ± 60	79 ± 41	0.16
Vitamin D (µg)	5 µg	3.6 ± 2.3	5.1 ± 3.5	0.09
Vitamin E (mg)	12 mg	14 ± 7	16.6 ± 9.7	0.41
Phosphorous (mg)	700 mg	1612 ± 763	1787 ± 942	0.64
Calcium (mg)	1000 mg	914 ± 387	1062 ± 548	0.34
Iron (mg)	7 mg	15 ± 11	16 ± 11	0.81
Selenium (µg)	55 µg	67 ± 30	79 ± 43	0.49
Zinc (mg)	10 mg	12 ± 6	13 ± 7	0.58
Sodium (mg)	1600 mg	2983 ± 1559	3385 ± 1964	0.59
Potassium (mg)	2000 mg	3798 ± 1603	4015 ± 1958	0.85
Magnesium (mg)	375 mg	359 ± 200	343 ± 189	0.67
Copper (mg)	1 mg	1.2 ± 0.6	1.5 ± 0.8	0.31
Chloride (mg)	800 mg	4407 ± 2407	4885 ± 2886	0.64
Manganese (mg)	2 mg	3.1 ± 1.5	3.2 ± 1.8	0.85
Iodine (µg)	15 µg	169 ± 76	201 ± 100	0.28

**Table 3:** Change in food group intake between pre-course and post-course time points, obtained from analysis of food frequency questionnaires. (*p*-values reaching statistical significance are in bold and accompanied by an asterisk).

Food Group	Pre-Course	Post-Course	*p*-Value
Red meats	0.62 ± 0.35	0.85 ± 0.56	0.07
Processed meats	0.58 ± 0.77	0.33 ± 0.27	0.08
Poultry	0.31 ± 0.26	0.25 ± 0.22	0.73
Organ meats	0.04 ± 0.07	0.11 ± 0.09	0.01 *
Fish	0.55 ± 0.43	0.68 ± 0.49	0.04 *
Fried foods	0.21 ± 0.14	0.30 ± 0.16	0.03 *
Refined carbohydrates	0.84 ± 0.56	1.28 ± 1.17	0.26
Whole grains	0.66 ± 0.41	0.95 ± 0.75	0.29
Cereal	0.69 ± 1.45	0.47 ± 0.55	0.76
Potatoes	0.34 ± 0.26	0.49 ± 0.34	0.06
Pasta meals	0.42 ± 0.31	0.34 ± 0.25	0.66
High-fat dairy products	2.03 ± 1.15	3.59 ± 3.07	0.09
Low-fat dairy products	0.21 ± 0.27	0.36 ± 0.28	0.02 *
Egg dishes	0.61 ± 0.49	0.43 ± 0.29	0.34
Fruit	2.02 ± 1.2	1.38 ± 0.84	0.04 *
Green leafy vegetables	0.77 ± 0.42	1.25 ± 1.03	0.11
Cruciferous vegetables	0.68 ± 0.61	0.44 ± 0.32	0.34
Starchy vegetables	0.42 ± 0.56	0.37 ± 0.24	0.16
Other vegetables	3.98 ± 2.17	3.72 ± 2.03	0.66
Legumes	0.30 ± 0.24	0.22 ± 0.25	0.10
Sweets	1.77 ± 1.29	2.45 ± 2.00	0.31
Snacks	0.48 ± 1.00	0.37 ± 0.66	0.40
Soups	1.15 ± 1.24	1.11 ± 1.17	0.48
Sauces	0.19 ± 0.17	0.27 ± 0.28	0.31
Condiments	2.68 ± 1.65	3.17 ± 2.12	0.58
Non-alcoholic beverages	2.39 ± 1.71	1.99 ± 1.42	0.45
Alcoholic beverages	1.43 ± 1.21	1.42 ± 1.29	0.91
Fruit Juice	0.42 ± 0.71	0.39 ± 0.53	0.50
Sweetened beverages	0.81 ± 1.03	0.78 ± 0.74	0.56

**Table 4:** Change in participants consumption of milk and dairy products between pre-course and post-course time points, obtained from analysis of food frequency questionnaires.

Dairy Intake	Pre-Course	Post-Course	*p*-Value
Total Milk (mL)	177 ± 120 mls	192 ± 134 mls	0.6
High-fat dairy products (servings/day)	2.03 ± 1.15	3.59 ± 3.07	0.09
Low-fat dairy products (servings/day)	0.21 ± 0.27	0.36 ± 0.28	0.02 *
Total dairy products	2.24 ± 1.23	3.35 ± 3.16	0.07
Unpasteurised milk (mL)	23 ± 116	239 ± 51	<0.0001 *
Unpasteurised dairy products (servings/day)	0.01 ± 0.04	1.2 ± 1.4	<0.0001 *

(*p*-values reaching statistical significance are in bold and accompanied by an asterisk).

**Table 5:** Short-chain-fatty-acid (SCFA) concentrations; pre- and post-course results.

SCFA (µmol/g)	Pre-Course Mean (SD)	Post-Course Mean (SD)	*p*-Value
Acetate	27.0 (8.6)	29.3 (10.3)	0.268
Propionate	14.0 (7.0)	16.3 (7.6)	0.091
Iso-butyrate	2.4 (1.1)	2.6 (0.9)	0.485
Butyrate	17.6 (9.6)	19.0 (10.8)	0.156
Iso-valerate	3.2 (1.9)	3.4 (1.7)	0.498
Valerate	2.3 (0.8)	2.5 (0.8)	0.049 *
Total BCFA	5.6 (2.9)	6.0 (2.5)	0.44
Total SCFA	66.6 (24.7)	73.1 (27.7)	0.113

*p*-values reaching statistical significance are in bold and accompanied by an asterisk. SD: Standard deviation.

**Table 6:** Results of psychological scales at pre- and post-course time points.

Scale	Pre-Course Mean (SD)	Post-Course Mean (SD)		*p*-Value
PSS	14.96 (6.23)	13.13 (5.12)		0.149
HADS-A	5.61 (3.72)	4.83 (3.21)		0.274
HADS-D	3.04 (2.82)	3.83 (3.42)		0.198
HADS-T	8.65 (5.37)	8.65 (5.93)		1
**Subgroup Analysis**	**Pre-Course Mean (SD)**	**Post-Course Mean (SD)**	**Change (Delta) between Pre- and Post-Course Scores Mean (SD)**	***p*-Value ^†^**
PSS-Highest	19.58 (3.12)	15.17(5.15)	−4.42 (5.9)	0.026 *
PSS-Lowest	10.08 (4.42)	10.91 (4.25)	1.0 (4.58)	
HADS-A-Highest	7.67 (2.85)	5.56(3.44)	−2.11 (2.30)	0.004 *
HADS-A-Lowest	1.75 (1.17)	4 (2.35)	2.25 (2.30)	

HADS: Hamilton Anxiety and Depression Scale, HADS-D: HADS-Depression subscale, HADS-A: HADS-Anxiety subscale, HADS-T: HADS-Total score, PSS: Perceived Stress Scale, SD: Standard deviation. PSS-highest refers to those participants with scores above the median and PSS-lowest to those with scores below the median. HADS-A-highest refers to those participants with scores above the median and HADS-A-lowest, to those with scores below the median. († the *p*-value in this case refers to the comparison between the mean scores of delta PSS-highest and delta PSS-lowest as well as the mean scores between delta HADS-A-highest and delta HADS-A-lowest. *p*-values reaching statistical significance are in bold and accompanied by an asterisk).
